# Multifaceted Impact of Lipid Extraction on the Characteristics of Polymer-Based Sewage Sludge towards Sustainable Sludge Management

**DOI:** 10.3390/polym16182646

**Published:** 2024-09-19

**Authors:** Nor Afifah Khalil, Ahmad Fiqhri Lajulliadi, Fatin Najwa Joynal Abedin, Ahmad Noor Syimir Fizal, Sairul Izwan Safie, Muzafar Zulkifli, Wirach Taweepreda, Md Sohrab Hossain, Ahmad Naim Ahmad Yahaya

**Affiliations:** 1 Polymer Science Program, Division of Physical Science, Faculty of Science, Prince of Songkla University, Hat-Yai, Songkla 90110, Thailand; 6510230020@email.psu.ac.th; 2 Malaysian Institute of Chemical and BioEngineering Technology, Universiti Kuala Lumpur, Alor Gajah 78000, Melaka, Malaysia; fiqhri.lajulliadi26@s.unikl.edu.my (A.F.L.); fatin.joynal28@s.unikl.edu.my (F.N.J.A.); 3 Centre for Sustainability of Mineral and Resource Recovery Technology (SMaRRT), Universiti Malaysia Pahang Al-Sultan Abdullah, Lebuh Persiaran Tun Khalil Yaakob, Gambang 26300, Pahang, Malaysia; syimirahmad@gmail.com; 4 Plant Engineering Technology Section, Malaysian Institute of Industrial Technology, Universiti Kuala Lumpur, Masai 81750, Johor, Malaysia; sairulizwan@unikl.edu.my; 5 Green Chemistry and Sustainability Cluster, Malaysian Institute of Chemical and BioEngineering Technology, Universiti Kuala Lumpur, Alor Gajah 78000, Melaka, Malaysia; muzafar@unikl.edu.my; 6 HICoE-Centre for Biofuel and Biochemical Research, Institute of Sustainable Energy and Resources, Fundamental and Applied Sciences Department, Universiti Teknologi PETRONAS (UTP), Seri Iskandar 32610, Perak, Malaysia; sohrab.hossain@utp.edu.my

**Keywords:** sewage sludge, dewatered sludge, polymer-based sludge, extraction, lipid extraction, sludge characterization, bibliometric analysis, sustainable sludge management

## Abstract

Dewatered sludge (DS) is a sewage sludge with a unique property due to extracellular polymeric substances (EPSs) and polymer flocculants. These components form a stable 3D polymer network to increase dewatering efficiency, leaving behind valuable materials such as lipids. This article explored the influences of DS particle size on lipid yield and the effects of extraction on the chemical, morphological, and thermal properties of the residual dewatered sludge (RDS). Lipid yields with unimodal distribution were observed across the particle size ranges (<0.5, 0.5–1.0, 1.0–2.0, 2.0–4.0, and 4.0 mm). The highest lipid yield of 1.95% was extracted from 1.0–2.0 mm after 4 h at 70 °C and 0.1 g/mL sludge-to-solvent ratio. Efficiency was influenced by the DS’s morphology, facilitating solvent infiltration and pore diffusion. The extraction process reduced water and organic fractions, resulting in higher thermal stability. Bibliometric analysis of “extraction*” and “sewage sludge” shows increasing research interest from 1973 to 2024. Five research clusters were observed: heavy metal speciation and stabilization, sludge and its bioavailability, extraction techniques and resource recovery, contaminants remediation, as well as phosphorus recovery and agricultural applications. These clusters highlight the diverse approaches to researching DS and RDS while promoting sustainable waste management.

## 1. Introduction

Wastewater treatment plants around the world produce large amounts of sludge annually as waste, with a generation rate of 0.1 to 30.8 kg/cap·day [1,2,3]. China alone generates around 20 to 30 million metric tons of sewage sludge each year via the operation of more than 3000 municipal wastewater treatment facilities throughout the country [2,4]. Germany, Spain, France, and the United Kingdom produced the highest sludge among the European countries in 2020, with 1749.86, 1210.40, 1174.00, and 1134.70 thousand metric tons of sludge [5].

Depending on the treatment processes, wastewater and sludge treatment plants produce various types of sewage sludge such as primary, secondary, dewatered, or stabilized sludge. A conventional wastewater treatment plant receives the influent of raw sewage into the preliminary treatment to remove large particles by settling and retaining most of the suspended organic material in the tank [2]. The large particles, such as sand, grit, and stones, sink to the bottom of the holding tank for disposal [2]. The next stage is the primary treatment of the settling process by a primary clarifier, settler, or sedimentation after the physicochemical process [6,7,8]. Primary treatment is aimed at removing scum, grease, and oil from sewage through skimming, along with the production of sludge by settling before it is removed as the primary sludge [2,9]. The primary sludge consists of a combination of floating grease, watery solids, and organic materials [2,10]. The remaining sewage from the primary treatment is transferred to the secondary treatment. Secondary treatment is a biological process aimed at removing suspended solids as the secondary sludge or activated sludge [2,6,7,8,9]. The secondary sludge is composed of water, suspended solids of inorganic particles, microbial cells, and extracellular polymeric substances (EPSs) [6,11,12,13]. EPSs are the product of active secretion, cell surface material shedding, cell lysis, and sorption from the environment [12]. The presence of cations such as Ca^2+^, Cu^2+^, Mg^2+^, and Fe^3+^ in the sewage neutralized the net negative charge of EPSs and further reduced the negative surface charge of the anionic groups of EPSs as well as the negatively charged particles of microbial cells, which then initiated bridging effects resulting in the destabilization and aggregation of flocs [11,12,14]. The flocs settled in the tank as secondary sludge or activated sludge [11,12]. The secondary sludge can be further treated by either aerobic or anaerobic digestion processes at a wastewater treatment plant with an additional setup, producing digested sludge [6,15]. Conventionally, the secondary sludge will undergo thickening and conditioning with the addition of polymer flocculant and a subsequent dewatering process to produce dewatered sludge (DS). The addition of flocculant such as cationic polyacrylamide (CPAM) increases the solid content of the conditioned sludge up to 2–5% [15]. The CPAM concentration in the conditioned sludge varied from 0.5 to 5 g/kg of total solids (TSs) [15]. The polymer flocculant reacted with the flocs of the secondary sludge by adsorption and bridging mechanisms, forming a 3D floc network with pores and channels [9]. The 3D floc network of sludge with hydrophilic functional groups traps water and soluble EPSs in their interstitial spaces [16]. The 3D floc network also acted like a membrane, assisting in the dewatering process by squeezing out water and some soluble EPSs from their interstitial spaces [14,17], resulting in the generation of DS. In China alone, more than 80% of DS as the end result of wastewater treatment needs to be treated further or disposed of [18].

The composition and properties of DS differ compared to other types of sewage sludge due to the inclusion of a polymer flocculant. The characteristics of various sludges are presented in Table 1. Generally, DS has around 73 to 83% water content compared to 98% of wet primary or secondary sludges [2]. The addition of polymer flocculant such as CPAM also increased the solid content in DS to 12–40% compared to 2–12% of primary sludge, secondary sludge, or waste-activated sludge (WAS) [19,20,21]. The composition of sewage sludge, particularly WAS and DS, are presented in Table 2, where they consist of EPSs with 50–80 wt.% of a total organic fraction and is mainly composed of proteins, polysaccharides, humic acid substances, and lipids [11,22,23]. A review by Wu et al. [23] mentioned that DS can be treated by anaerobic fermentation and simultaneously recover carbon, nitrogen, and phosphorus. However, inhibition of methanogen activity might occur due to the high solid content and the possibility of volatile fatty acid buildup [23]. Alternatively, sludge that is readily available for free or at a significantly lower cost can be used sustainably to recover lipid and create a value-added product [24,25]. The recovered lipids can be converted to fatty acid methyl ester (FAME), where the C8–C23 fraction can be used as biodiesel, while the C8–C16 fraction can be further explored as a sustainable aviation fuel (SAF) [7,26,27].

Lipids from sewage sludge are mainly recovered by solvent extraction based on solid–liquid extraction methodology [24,32]. Table 3 shows the extraction methods and solvent used in the extraction of lipids. Advanced technologies—namely, supercritical fluid extraction, microwave-assisted solvents, accelerated solvent extraction system, and liquefied dimethyl ether—managed to extract around 0.65 to 27.43% of lipids compared to the conventional methods. These advanced technologies require specialized equipment and setup, leading to the abandonment of techniques [33,34,35,36,37]. Supercritical fluid extraction (SFE) technology exploits the characteristics of fluids at their critical pressure and temperature in order to change the density of the fluid and increase the analyte solubility [37]. SFE requires a special setup with pumps and fluid supply, a heated oven with extraction cells, restrictors, and collection devices to obtain the intended pressure and temperature [37]. On the other hand, conventional solvent extraction methods such as Soxhlet extraction, the modified Bligh and Dyer method, acid hydrolysis, water bath shaking, and boiling extraction produced 1.0 to 40.21% of lipids. Soxhlet extraction adopted a simple methodology with a less complex setup and minimal training, making it a cheaper and more suitable option for recovering valuable material from the sludge [38]. The Soxhlet methodology has been the standard leaching technique and the primary reference for new development or modification techniques [38]. The method requires an extraction setup with a siphon mechanism, in order to bring the fresh solvent into contact with the sample for a few cycles by employing the solvent’s boiling point [38,39]. Soxhlet extraction is categorized as a batch system; nevertheless, given that the extractant is recirculated through the sample, the system has a continuous characteristic [40]. Soxhlet extraction is a well-established technique with good reproducibility and efficiency [41], especially in the extraction of lipids from sewage sludge with up to 2.5–40% yield [4,7,29,32,42,43]. Furthermore, lipids extracted by Soxhlet method show low antioxidant activity compared to the Folch method [44]. Various polar and non-polar solvents are utilized in the extraction technique for the extraction and separation of lipids from various oil matrices [23,36,37]. The nature of the solvent has a significant impact on the type of lipids in the extract [44]. Polar solvents (bromopropane, ethanol, and methanol) removed more polar lipids than non-polar solvents (n-hexane or hexane), while mixed solvents (chloroform–methanol or methanol–hexane–acetone) showed greater extraction efficiency than single solvents [7]. The usage of polar solvents might lead to the extraction of polar compounds such as proteins, carbohydrates, and phosphatides, beside triacylglycerols (TAGs) [45]. Hexane is a non-polar solvent that is viewed as the most promising solvent to extract the neutral lipid of TAGs for the production of biodiesel [44].

The efficiency of the Soxhlet extraction process as a solid–liquid extraction is influenced by the medium and matrix parameters [52]. Most of the past research utilizing sewage sludge explored Soxhlet extraction based on medium parameters such as temperature within 60–90 °C [7,29,32], extraction time of 2–8 h [7,25,29,32,43,50,53,54], and cosolvent [7]. Numerous studies were conducted on matrix parameter of sludge-to-solvent ratio (S/L) [7,25,29,43,53,54]. Particle size as a matrix parameter, however, has not received much attention [51,52]. Despite the accomplishments of various research projects in the extraction of lipids from sludge, the fate of residual sludge is scarcely studied [55]. The extraction process is expected to reduce the amount of sludge to be managed, besides altering the characteristics of the residual sludge. Hence, this paper aimed to study the effect of different particle sizes of DS on the yield of lipids. This paper further explores the multiple impacts of the extraction process on the chemical composition, morphology, elemental composition, and thermal characteristics of residual dewatered sludge (RDS). Hence, this research also aims to provide insight into possible sustainable management options for RDS.

## 2. Materials and Methods

### 2.1. Material and Reagent

DS, WAS, and polymer flocculant were obtained from a sludge treatment plant. Hexane Exxsol^®^ (ExxonMobil, Spring, TX, USA) was used in the extraction experiment.

### 2.2. Preparation of Sludge Sample

The DS utilized in this study was obtained from a sludge treatment plant that generates WAS. The WAS was added with polymer flocculant consisting of polyacrylamide (PAM) during the conditioning process. A decanter was used in the dewatering process of the sludge mixture to produce DS. The DS was kept in a sludge compartment prior to disposal. The DS was collected by shovel from the sludge compartment and placed in a closed container for transport to the laboratory, while WAS was collected directly from the sludge outlet of the secondary treatment. The WAS and polymer flocculant were placed in an individual container. The DS, WAS, and polymer flocculant were transported to the laboratory for further treatment and analysis. MC, TS, and VS of DS were analyzed based on method 2540 G [54]. The characteristics of the DS are presented in Table 4.

The DS was mixed, spread on a tray, and dried in an oven UM300 (Memmert, Schawabach, Germany) at 60 °C until it reached a constant moisture content. The dried DS was ground up using a pestle and mortar for further testing and analysis. The dried DS was sieved using a mechanical sieve EFL2000 (Endercotts, Hope Valley, UK) and the particle size distribution was analyzed based on sieve analysis for size ranges of <0.5 mm, 0.5–1.0 mm, 1.0–2.0 mm, 2.0–4.0 mm, and >4.0 mm. The particle distribution of the DS is shown in Table 5. Moisture content was analyzed by a moisture analyzer HR-250AZ (A&D, Tokyo, Japan) prior to the lipid extraction experiments. The WAS was placed in jars for sedimentation and decantation. The upper layer was decanted, leaving the bottom layer for further drying. Drying was carried out in an oven UM300 (Memmert, Schawabach, Germany) at 105 °C until constant weight. The WAS was grounded using a pestle and mortar prior to further analysis.

### 2.3. Extraction and Separation of Lipids

Lipids were extracted from sludge samples by Soxhlet extraction in duplication for each particle size range of <0.5, 0.5–1.0, 1.0–2.0, 2.0–4.0, and >4.0 mm; 200 mL of hexane was filled up in each of the 250 mL round bottom flasks (Favorit, Milan, Italy) and placed on the six-pot heating mantle (Biobase, Jinan, Shandong, China); 20 g of DS of various size ranges was filled up in a cellulose thimble CT30100 (Favorit, Milan, Italy) and placed in the extraction chamber of the Soxhlet extractor. The Soxhlet extractor was then connected to the flask and condenser. The condenser was previously connected to a chiller TC501D (Brookfield, MA, USA) via tubing for cooling water circulation. The cooling water temperature was set at 20 °C. The extraction process was conducted at a constant temperature of 70 °C with a S/L of 0.1 g/mL for 4 h. The temperature of 70 °C is slightly higher than the boiling point of hexane, allowing for efficient extraction without accelerating the solvent’s evaporation, thus maintaining system stability throughout the process. A minimum extraction time of 4 h was used, ensuring sufficient interaction between the solvent and sludge particles, and balancing practicality and efficiency for further extractant and residual analysis. Upon completion of the extraction process, any remaining resultant mixture in the extraction chamber was carefully removed and placed in the flask. The flask containing resultant mixture was subsequently cooled for an hour in a desiccator prior to separation and further analysis. The RDS was air-dried at ambient temperature prior to further analysis.

### 2.4. Separation of Lipids

Separation of lipids from the resultant mixture was conducted by evaporation using a rotary evaporator Rotavapor R-200 (Büchi, Uster, Switzerland) and assisted by a vacuum pump. The flask containing the resultant mixture was connected to the rotatory evaporator and placed in the water bath for solvent evaporation. The lipids contained in the flask as the product of evaporation were then cooled in a desiccator and weighed. The evaporation, cooling, and weighing were repeated until a constant weight of lipids was obtained.

### 2.5. Lipids Yield Analysis

The lipid yield was determined based on the total mass of dry sludge [29,56] and calculated using Equation (1).

(1) $$Lipids\;yield\left(\%\right)=\frac{Lipids\;mass\left(g\right)}{Sludge\;solid\;mass\left(g\right)}\times 100\%$$

### 2.6. Characterization of DS, WAS, and Polymer Flocculant

An attenuated total reflectance Fourier transform infrared (ATR-FTIR) spectrometer Nicolet^TM^ (Thermo Fisher Scientific, Waltham, MA, USA) system equipped with an ATR diamond crystal was used to obtain FTIR spectra of the DS, RDS, WAS, and polymer flocculant. The analysis was conducted based on 26 scans at 8.0 cm^−1^ resolution in the range of 4000–400 cm^−1^. Omnic software (Thermo Fisher Scientific, Waltham, MA, USA) was used for the FTIR spectrometer operation and spectra collection.

The morphological analysis of the DS and RDS were conducted using scanning electron microscopy (SEM) of the TM4000Plus II (Hitachi, Tokyo, Japan). The elemental analysis was also conducted based on area mapping utilizing energy-dispersive X-ray spectroscopy (EDS) and Aztech software 6.1 (Oxford Instrument, High Wycombe, UK) [57,58].

Thermogravimetric analysis was conducted for the DS, RDS, WAS, and polymer flocculant using a thermogravimetric analyzer (TGA) (Mettler Toledo, Columbus, OH, USA). The analysis was performed at a temperature of 30–900 °C with 70 mL/min of protective gas (N_2_) at a 10 °C/min increment. Purge gas was introduced at 900–1000 °C at 20 mL/min.

### 2.7. Bibliometric Analysis

Bibliometric analysis was conducted based on data retrieved from the Scopus database. The data were obtained on 10 June 2024 using search strings of (“extraction*”) AND (“sewage sludge”) based on article title, abstract, and keywords. The data were analyzed and visualized using MS Excel version 2408 (Microsoft Office, Redmond, WA, USA) and VOSviewer Version 1.6.20 (Leiden University, Leiden, The Netherlands) [59,60]. Science mapping was used specifically on the co-occurrence analysis. A total of 252 author keywords were used as the unit of co-occurrence analysis based on the full counting method.

## 3. Results

### 3.1. DS as a Raw Material in Lipid Feedstock for Biodiesel

The overall biodiesel production cost is influenced by the feedstock price and production costs. The significant economic issue of producing biodiesel is always associated with the high price of feedstock, which accounts for up to 80% of the entire operation cost [61]. Sewage sludge can be utilized in the extraction of lipids as part of the energy recovery in the sludge management scheme [55]. The extracted lipid can be used as feedstock for biodiesel production [25]. The high cost associated with feedstock is minimized by the use of sewage sludge as the raw material to produce oil compared to edible oil [61]. The estimated production cost of USD 3.11–3.23 per gallon of biodiesel from the primary and secondary sludges as raw material is lower compared to refined soy (USD 4.00–4.50) [61].

A preliminary evaluation of the utilization of sewage sludge of secondary sludge in biodiesel production was conducted by Dufreche et al. (2007) based on the operational cost. The production of biodiesel poses challenges, especially in operation and maintenance (O&M), which make up around 85% of the total operational cost due to the high liquid volume of the secondary sludge [48]. The liquid content needs to be reduced by centrifugation and drying to obtain the 0.8–12.0 wt.% solid fraction for subsequent extraction processes. Alternatively, using DS with 66–80% moisture content is predicted to result in a 13.8% decrease in O&M costs since it eliminates the requirement for centrifugation and only requires drying to produce 17–15% of the solid fraction’s weight. Other O&M costs such as the extraction and biodiesel processing costs can be optimized further based on process parameters. Parameters such as temperature and time of the extraction process can also be optimized to obtain high yields [52]. However, balancing optimal cost and yield in process optimization is challenging since the parameters that minimize cost often differ from those that maximize yield [52]. Despite the challenges, the success of solvent extraction strongly depends on the matrix condition, particularly the particle size of the DS. Hence, the influence of DS particle size is investigated in the subsequent section.

### 3.2. Lipid Extraction from DS

**Figure 1:**
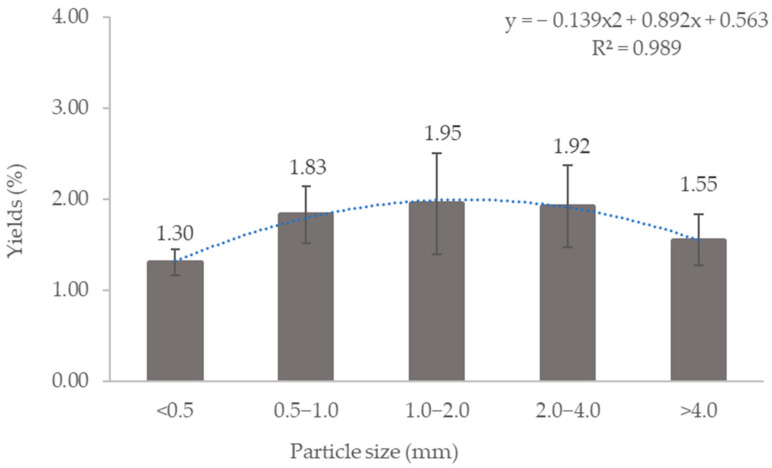
The yield of lipids extracted from DS from various particle size range at 70 °C, 4 h, and 0.1 g/mL S/L.

The efficiency of the extraction process depends on various critical variables, including particle size [62]. The particle size of the sludge is one of the important variables influencing the rate of extractable lipids and can be modified as an effective extraction size to regulate the amount of lipids produced [63]. There are five effective extraction sizes tested based on particle size ranges of <0.5, 0.5–1.0, 1.0–2.0, 2.0–4.0, and >4.0 mm. Figure 1 shows the yield of lipids extracted from various particle sizes at 70 °C with a S/L of 0.1 g/mL for 4 h. The graph shows a unimodal distribution of lipid yield across the particle size ranges. The lowest yield of lipids, 1.30 ± 0.14%, is extracted from the smallest particle size of <0.5 mm. The lipid yield increased to 1.83 ± 0.31% with the increase in particle sizes (0.5–1.0 mm). The highest lipid content of 1.95 ± 0.56% was obtained from particle size 1.0–2.0 mm. Further increases in particle sizes to 2.0–4.0 mm and >4.0 mm resulted in a decrease in lipid yield to 1.92 ± 0.45% and 1.55 ± 0.28%, respectively. Possible factors influencing the lipid yield from DS of various particle sizes are further discussed in the next paragraph.

The extraction of lipids from matrices is a mass transfer process, which can be accomplished either by directly releasing bulk lipids while disrupting the matrices’ structure or by diffusion of lipids over the structure [44]. However, diffusion is the primary mass transfer mechanism in the extraction that is influenced by the porosity and pore tortuosity of the matrices [44,64,65], specifically the DS in this project. The DS in this study had a dense structure with pores and gaps, where increasing the total size from <0.5 by 0.5–1.0 to 1.0–2.0 mm resulted in more pores, which increased the surface area and further promoted considerable mass transfer of solute between phases via pore diffusion [44,64,66]. The decrease in the lipid’s yield for a larger particle size of the DS might be due to the tortuosity of the matrices [44]. The structure of the DS particles also changed in the drying process [67]. The relationship between the particle size of the DS and the lipid yield will be further discussed based on the morphological analysis presented in Section 3.4.

**Figure 2:**
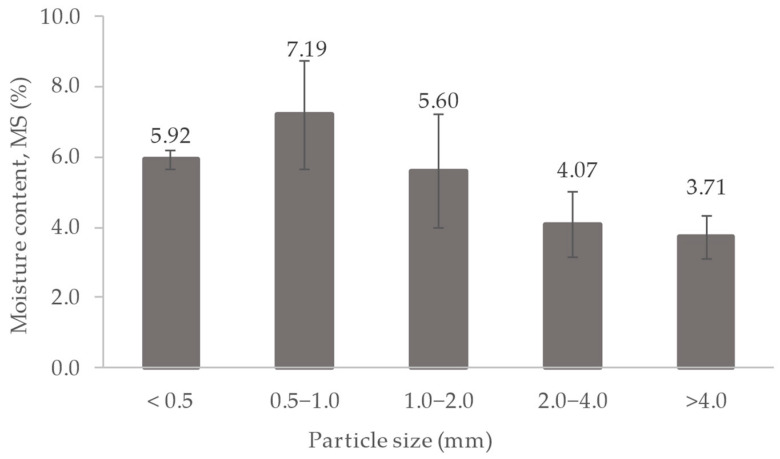
Moisture content of DS.

Moisture content of sludge is also one of the possible factors that is closely related to the extractant in determining the efficiency of the extraction process [68]. Water contained in the surroundings and within the DS particle interferes with the extraction process by inhibiting the good penetration of solvent inside the solid sludge particle [68]. Figure 2 shows the moisture content of DS in various particle size ranges. The smaller size of the DS (<0.5 mm and 0.5–1.0 mm) has higher moisture content (5.92 ± 0.26% and 7.19 ± 1.55%) due to its high water retention capacity [67], resulting in lower lipid yield. In contrast, larger sizes (1.0–2.0, 2.0–4.0 and >4.0) exhibit lower moisture content (5.60 ± 1.60%, 4.07 ± 0.95%, and 3.71 ± 0.60%) due to the larger pore diameter, which decreases the water retention capacity [67], thus resulting in higher lipid yield.

The extraction process successfully extracted lipids from the DS with the highest yield of 1.95 ± 0.56% at 70 °C for 4 h with a S/L of 0.1 g/mL lipids extracted from DS have a lower lipid yield relative to the total mass of the sludge as compared to primary (20.34–40.21%) and secondary sludges (4.6–27.43%), as presented previously in Table 3. This is due to variations in the composition of DS with lower volatile solid content of 46.75 ± 0.74% compared to 59–88% of primary and secondary sludge. Lower volatile solid content reflects lower lipid composition in the DS. Primary and secondary sludge mainly consist of organic matters such as fat, grease, microbial cells, and silicate minerals [69], while the DS consists of additional polymer flocculant forming a 3D floc network between the sludge, polymer flocculant, and organic matter, thus increasing the total solid content. The extraction process by hexane as solvent at 70 °C succeeded in diffusing lipids into the solvent while loosening and disaggregating the surface particles. Up to 6.9% of lipids are recovered from the DS, relative to the 27.97% total organic content.

### 3.3. FTIR Analysis of DS and RDS

FTIR analysis was conducted to identify the chemical composition of DS with WAS and polymer flocculant as reference, offering important insights into the composition and interactions within the sludge matrix.

**Figure 3:**
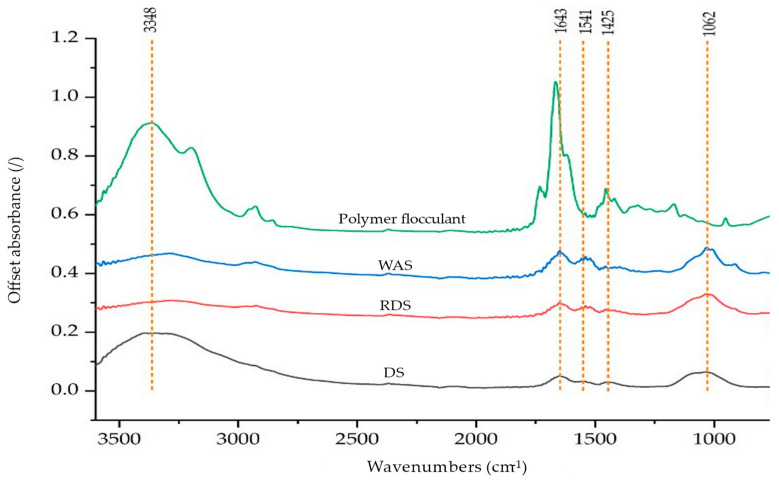
FTIR spectra of DS, RDS, WAS, and polymer flocculant.

FTIR of DS shows a unique spectrum with peaks corresponding to the specific functional groups as presented in Figure 3. The FTIR of DS shows five broad and distinct peaks where a peak was identified within the single-bond region (4000–2500 cm^−1^), two peaks in the double-bond region (2000–1500 cm^−1^), and two peaks in the fingerprint region (1500–600 cm^−1^). A broad peak at 3348 cm^−1^ within the single bond region corresponds to the OH stretching of hydroxyl groups (3570–3200 cm^−1^) of acids or alcohols [70,71]. A peak at 1643 cm^−1^ was observed, similar to the characteristic peaks of WAS and polymer flocculant. The peak corresponded to primary amides of peptide and protein originated from WAS [70,72,73] or C=C vibration of the olefinic compound within the polymer flocculant [71]. A peak at 1541 cm^−1^ was also observed, comparable to the peak of WAS that corresponds to ${NH}_{3}^{+}$ bond in protein [73]. The next peak of the DS was detected at 1425 cm^−1^ attributable to C-H methylene (CH_2_) of the polymer flocculant of PAM [71].

**Figure 4:**
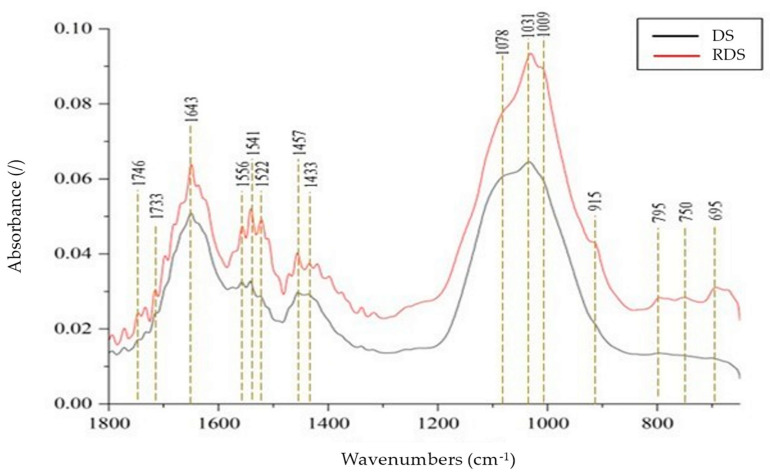
FTIR of the DS and RDS at 650–1800 cm^−1^.

A broad peak at 1031 cm^−1^ could be due to O-C-C stretch within 1100–1030 cm^−1^ of the ester [72,74]. Ester can be identified using the rule of three, with three intense peaks within the 1750–1735 cm^−1^ (C=O), 1210–1160 cm^−1^ (C-C-O), and 1100–1031 cm^−1^ (O-C-C) [74,75]. Further evaluation was conducted within the region 1800–800 cm^−1^ and is presented in Figure 4. The peak at 1746 cm^−1^ was seen to be attributed to carbonyl compounds; however, a broad peak was observed at 1210–1160 cm^−1^ due to the overlapping of peaks within the 1200–900 cm^−1^ region. Multiple peaks at 1078 cm^−1^ and 1031 cm^−1^ were observed within the same region due to the O-C-C stretching vibration of ester [74,75] and stretching vibration of the CO bond of the primary alcohol in saccharide, which originated from WAS [72,73]. Notably, a peak at 1733 cm^−1^ was observed due to the carbonyl bond of PAM [72,75,76,77] and the carboxylic acids of fats in DS [72,73,75]. Possible overlapping of peaks at 1166 cm^−1^ and 953 cm^−1^ depicted by a broad spectra might be associated with C-C skeletal vibration and H vibration in C=C-H of PAM contained in the DS. De Oliveira Silva et al. [70] mentioned that the intense band around 1032 cm^−1^ within 1170–1000 cm^−1^ region is associated with the OH vibration of mineral compounds in the sludge.

Figure 3 and Figure 4 also present the FTIR spectra analysis used for RDS to determine the impact of the extraction process on the chemical composition. The FTIR spectra of the RDS appeared similar to the characteristic peaks of the DS, with no peak detected at 3348 cm^−1^, indicating no hydroxyl group. Observation of the FTIR spectra of RDS at 1800–650 cm^−1^ shows the appearance of peaks at 915, 795, 750, and 695 cm^−1^, as depicted in Figure 4. The peak at 915 cm^−1^ corresponds to the Si-O band of silicate ions, suggesting the presence of silicate minerals in the RDS [78,79]. Multiple peaks at 795, 750, and 655 cm^−1^ were also observed, attributed to C-H bending vibration associated with the aromatic ring absorption at 1650–1600 cm^−1^ [71]. The peak at 655 cm^−1^ paired with the intense and broad peak at 1130–1080 cm^−1^, possibly attributed to sulfate ions [71].

Overall, the DS consists of complex chemical compounds, as evidenced by the appearance of various FTIR peaks of functional groups related to amides, olefinic compounds, esters, saccharides, carbonyl bonds, and carboxylic acids, which originated from WAS and polymer flocculants [71]. The extraction process resulted in RDS with a similar chemical composition to DS, with defined peaks attributable to silicate minerals, aromatic compounds, and sulfate ions.

### 3.4. Morphology of DS and RDS

**Figure 5:**
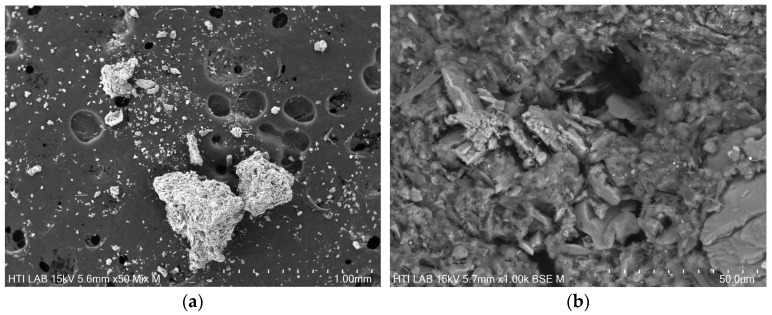
SEM analysis of (**a**) WAS at 50 magnification and (**b**) WAS at 1000 magnification.

The DS in this study was generated from WAS with the addition of polymer flocculant consisting of polyacrylamide. A previous morphological study shows the raw WAS flocs have a loosely packed structure when analyzed in freeze-dried form [80]. Figure 5a shows that upon drying and homogenization, the WAS exists in varied shapes and sizes. Plate-like aggregation can be seen on the surface of the particles as presented in Figure 5b [80,81,82,83].

**Figure 6:**
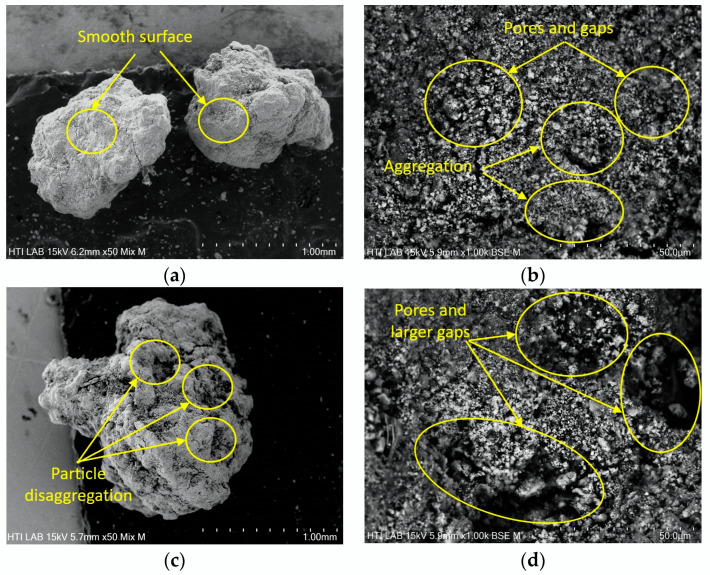
SEM image for particle size 1.0–2.0 of (**a**) DS at 50 magnification; (**b**) DS at 1000 magnification; (**c**) RDS at 50 magnification; (**d**) RDS at 1000 magnification.

The addition of polymer flocculant to WAS during the conditioning process and subsequent dewatering followed by drying led to the formation of DS with modified morphology [80]. Figure 6a depicts the initial observation of the DS morphology at 50 times magnification, where the surface appears smooth with a dense structure, demonstrating good cohesion and minimal porosity [84]. Further examination on the DS at 1000 magnification indicates that the dense structure consists of pores and gaps, with some aggregation as presented in Figure 6b [85]. The pores and gaps could be due to the interstitial spaces between the DS’s particles. Furthermore, the coexistence of the dense structure with pores and gaps also suggests the DS is compact and not completely impermeable. The 3D flocs network of the DS with pores and channels was formed due to the bridging between the anionic groups of EPS and negatively charged particles in the sludge, as well as the crosslinking of EPS with microbial cells [11,22]. The addition of polymer flocculant instilled adsorption and bridging effects, generating a larger 3D floc network [16]. While charge neutralization caused the aggregation of the sludge particles [84,85]. Aggregate flocs provide a cage effect, restricting bacterial and enzyme interaction with organic molecules and further resisting mass transfer and release of the organics in the flocs to the liquid phase [15]. Zhang et al. [17] analyzed the morphological changes of the raw sewage sludge that was being added with analytical-grade CPAM. They observed the morphology of the sewage sludge being altered from a dense surface to smooth with closed surface pores after CPAM addition. Abundant porous structures and channels are seen on the sewage sludge structure with the inclusion of skeletal builders such as sawdust or risk husk to CPAM and further enhance the dewatering process [17]. The dewatering process minimized the water content of the sludge, and further drying changed the structure of the sewage sludge [67], resulting in a denser structure with pores and gaps. The morphology of the DS in this research also differs from the sludge in the previous research by Olkiewicz et al. [43] where the untreated blended sludge used has large flocs with an incoherent morphological structure. While blended sludge flocs have a more compact structure and smaller size due to the disintegration process of acidification [43].

**Figure 7:**
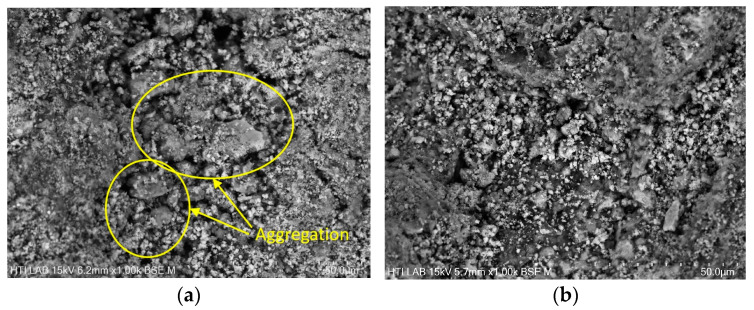

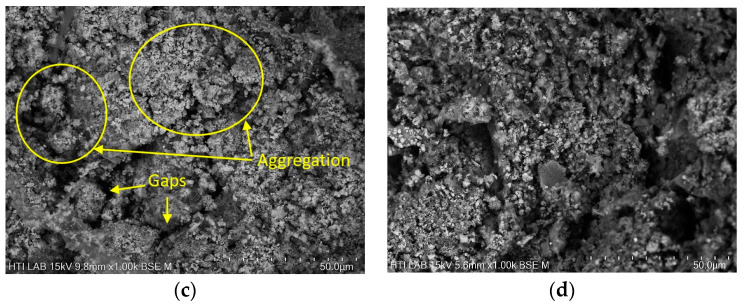
SEM analysis at 1000 magnification for (**a**) DS of <0.5 mm; (**b**) RDS of <0.5 mm; (**c**) DS of >4.0 mm; (**d**) RDS of >4.0 mm.

As discussed in Section 3.2, DS with particle sizes of 1.0–2.0 mm shows the highest lipid yield compared to smaller (<0.5 and 0.5–1.0 mm) and larger (2.0–4.0 and >4.0 mm) sizes. The extraction of lipids by diffusion is influenced by the morphology of DS. Figure 6b shows the DS with particle sizes of 1.0–2.0 mm consists of a higher number of pores and gaps with minimum aggregation compared to smaller particle sizes of <0.5 mm (Figure 7a. The pores and gaps allow for infiltration of solvent and enhance pore diffusion, hence increasing the yield of lipids [44,64]. The surface of smaller DS of <0.5 mm displays plate-like aggregation, impeding the efficient extraction of lipid. The plate-like aggregation on the DS is similar to the aggregation on the surface of WAS, as previously shown in Figure 5b. The increase in particle size from 1.0–2.0 mm to >4.0 mm resulted in more aggregation of smaller particles on its surface, as presented in Figure 7c. Larger particles exert more attractive forces on smaller particles, providing insight into the collective dynamics of the multiparticle system [86]. As clusters evolve into large-scale particles, their attractive forces become stronger and attract smaller clusters to aggregate into a single larger particle [86]. As a result, massive aggregation forms on the surface of the larger particle size, covering the pores.

Figure 6c,d also display the RDS with particle sizes 1.0–2.0 mm. The figure indicates that the extraction procedure did not alter the overall size of the sludge particles. However, the RDS structure was pitted and eroded due to disaggregation, evident by the appearance of an incomplete aggregate structure [87], as circled in Figure 6c. The extraction procedure also disrupted the surface of the 3D network of the sludge leading to disaggregation, thus exposing larger gaps and more pores as depicted in Figure 6d. More exposed gaps and pores further enhance diffusion for efficient mass transport of lipids by solvent. Similar observations were made on the morphology of RDS with particle sizes of <0.5 mm and >4.0 mm as depicted in Figure 7b,d, where the aggregation on the surface of the bigger particle is clearly seen.

### 3.5. Elemental Composition of DS and RDS

**Figure 8:**
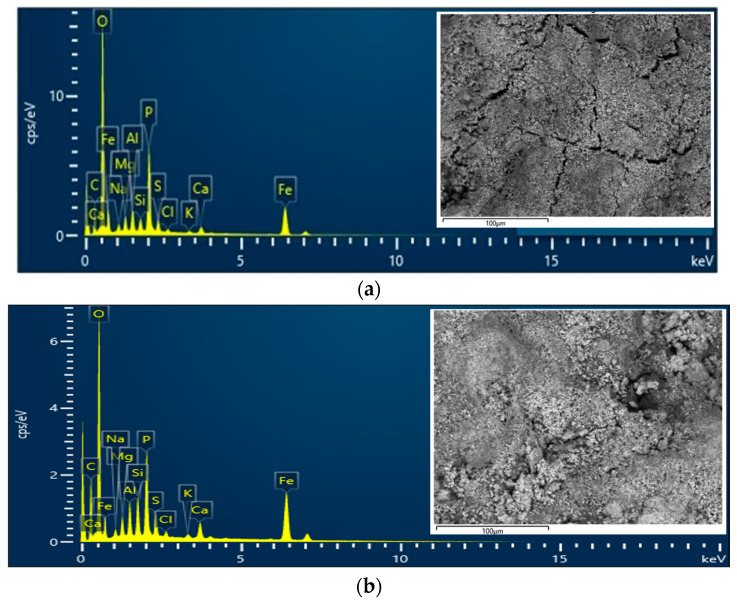
EDS and electron image of (**a**) DS and (**b**) RDS.

**Figure 9:**
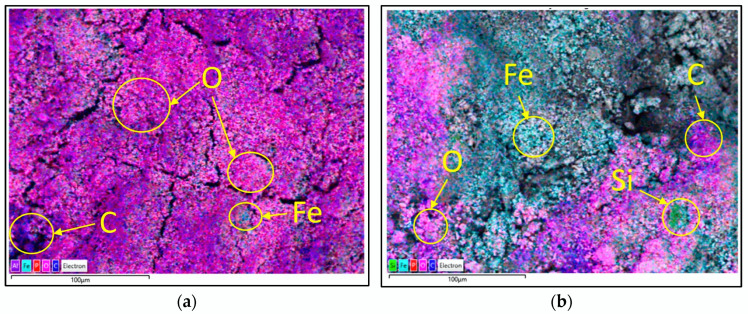
Distribution of elements on the surface of (**a**) DS and (**b**) RDS.

EDS analysis was performed to determine the effect of the extraction process on the DS. During the EDS measurement, different areas were scanned as presented in the electron image, and the corresponding peaks are shown in Figure 8. Figure 8a depicts the elemental analysis of the DS for the area shown in the electron image, with the even surface and gaps seen. The highest elements of O, C, Fe, and P, followed by Al, Mg, S, Ca, Si, Na, Cl, and K, were detected as presented in Table 6. Elements such as P, Si, Al, Fe, Ca, Mg, Na, and K are among the ash-forming elements present in relatively high amounts in the sewage sludge compared to Mg, Ca, and K [88]. A study by Detho et al. [89] found that sewage sludge contained the highest compounds of SiO_2_, Al_2_O_3_, and Fe_2_O_3_ at 49.3, 18.4, and 6.9%. The extraction process managed to reduce O, P, Al, Mg, and Na by 8.72, 2.32, 0.10, 0.16, and 0.28 wt.%. The reduction of elements is due to the disaggregation of smaller clusters that are shown by the uneven surface with gaps and aggregate, as depicted in the electron image in Figure 8b. The disaggregation also exposed the inner surface of that particular area, exposing elements such as Si and Fe. Figure 9 shows the distribution of elements on the surface of the dewatered DS and RDS based on area color mapping. As seen in Figure 9a, it is evident that O, denoted by the color pink, is evenly distributed throughout the surface of DS, with minimal appearance of C (purple) and Fe (light blue). Figure 9b shows the extraction process resulted in disaggregation on the surface of RDS, evident by the reduction of O and C, revealing Fe and Si (green).

### 3.6. Thermal Characteristics of DS and RDS

**Figure 10:**
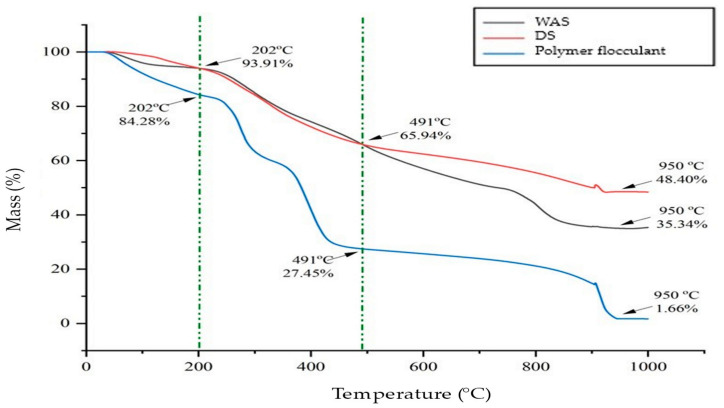
Thermal analysis of DS, WAS, and polymer flocculant.

Thermogravimetric analysis was undertaken to analyze the thermal characteristics of the DS and RDS by the mass loss trend during heating at temperatures ranging from 30 to 1000 °C [90]. Figure 10 shows the thermal degradation of DS from 30 to 1000 °C in comparison with WAS and polymer flocculant as the reference materials. It can be seen that thermal degradation occurred in three stages for the DS (30–202 °C, 202–491 °C, and 491–900 °C) and WAS (30–202 °C, 202–750 °C, and 750–900 °C), while there were four stages for polymer flocculant (30–210 °C, 210–350 °C, 350–450 °C, and 450–900 °C). Table 7 shows the mass loss of the DS, WAS, and polymer flocculant.

Stage 1 for DS and WAS started at 30–202 °C with a mass loss at of 3.52% and 5.27%, respectively. The mass loss below 150 °C is due to moisture evaporation [2,91,92] and is also associated with the evaporation of the light volatile materials [92,93,94]. The decreased mass loss of DS was caused by the removal of the free (bulk) and most of the interstitial water during the dewatering process and drying prior to analysis; hence, only vicinal water was left to be removed from the sludge’s surface by heat [2]. Thermal degradation of carbohydrate and lipids started in the temperature ranges of 110–420 °C and 150–515 °C, respectively [95].

Stage 2 mass loss trend of the DS is identical to the WAS from 202 to 491 °C and comparable to the polymer flocculant from 491 to 900 °C as presented by the two green lines. The plot reveals a decreasing trend in mass over the temperature range, which is caused by the physical transition or chemical interaction of components in the samples [92]. Thermal analysis of WAS and PAM (polymer flocculant) is detailed in research articles by Ghodke et al. [96] and Han et al. [97]. The DS shows a similar decreasing mass trend of WAS from 93.91% to 65.94% over temperature ranges of 202–491 °C, as seen by the crossing of these plots at both temperatures. The decreasing mass between 230 and 430 °C was caused by the combustion of organic compounds and residual organic components [93]. Some fractions of carbohydrate and lipids degraded within these temperature ranges, while proteins started to degrade at 210–310 °C [95]. Thus, 27.97% mass loss at 202–491 °C for DS and WAS was due to the thermal degradation of organic components (proteins and carboxyl groups) and carbon refractories (saturated aliphatic chains, aromatic rings, and N-alkyl long chain structures) [70]. The mass loss within this temperature range is also due to the fractions of polymer flocculant that exist in the DS, as evident by the increase in mass loss from 6.09 to 6.44% with the increasing temperature from 202 to 210, compared to 6.09 to 6.24% for WAS. Polymer flocculant, specifically PAM, started to undergo thermal degradation at 175–300 °C [98]. Thermal degradation of the polymer flocculant consisting of PAM involves the deamination process to form imides [97]. The decomposition of acryl amide (CO-NH) and trimethyl ammonium (N+(CH_3_)_3_) functional groups of PAM occurred between 192.59 and 354.97 °C [91]. Above 300 °C, specifically in the range of 308.9–444.77 °C, the decomposition of high-molecular polymers into low-molecular polymers occurs within DS [91]. The thermal degradation of polyacrylamide without free radicals takes place, causing substantial chain scission of a high-molecular-weight polymer, resulting in a low-molecular-weight polymer [77,91]. The thermal characteristics of DS at temperatures above 400 °C are also the results of the elimination of bound water [2,93]. The bound water that was formed by the hydroxides was released from the sludge–polymer floc network due to degradation and decomposition of the polymer, which resulted in changes in the flocs’ structure [2,93]. These TGA analyses were supported by the FTIR peaks detected at 1733 cm^−1^ and 1643 cm^−1^ attributed to the polymer functional group of the C=O carboxylic bond and the C=C of primary amide in DS [70,72,75,76,77,78].

Beyond 491 to 950 °C, the mass loss rate of the DS is lower compared to WAS and polymer flocculant. The mass loss pattern of the DS started to be different from WAS; however, the trend is comparable to the polymer flocculant. The higher residue within these ranges for DS is due to the formation of polyaromatic carbonaceous matter [97]. It is expected that flash pyrolysis at 600–900 °C will result in the formation of tar and coke from the carbon backbone [92,97]. This trend also suggested that the fraction of non-volatile compounds in DS is larger compared to both WAS and polymer flocculant. It is worth noting that there is a slight increase in weight at 910 °C due to the chemical reaction of the sludge compound with the sudden introduction of oxygen gas [92]. The mass decreased from 56.03% to 48.61% at 910 °C, with a mass loss of 7.42% due to the combustion of carbon black on gas switching from N_2_ to air [92]. Beyond 950 °C, only ash remained as the product of the combustion of carbon black [92]. The residual mass of DS is 48.40%, which is higher as compared to WAS and polymer flocculant (35.34% and 1.66%).

**Figure 11:**
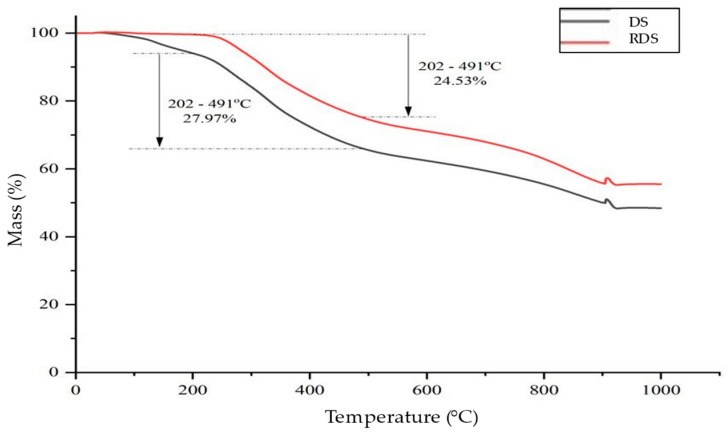
Thermal analysis of DS and RDS.

Figure 11 shows the thermal analysis conducted on RDS compared to DS. There are two steps in the thermal degradation of DS at low temperature: The first step is from 30 to 150 °C due to water removal and light volatile material evaporation [2,91,92,93,94]. The second step occurs at 150–202 °C, where biocompounds such as carbohydrate and lipids start to degrade [95]. It is apparent that the mass loss of RDS with the onset temperature shifted to a higher temperature of 83.55 °C indicates that some of the water and volatile compounds are eliminated from DS due to the extraction process, with a mass loss of 0.23% at 150 °C [92,93]. Lower mass loss (24.53%) within the temperature range of 202–491 °C for RDS compared to DS (27.97%) is due to the organic compound removed from the surface of the DS particles. The extraction managed to produce 1.95 ± 0.56% of the lipid fraction, while the remaining organic matter might be separated from the DS by disaggregation into small particles, as discussed in Section 3.4. Certain sludge treatment procedures can result in a reduction in organic content [99]. For example, anaerobic digestion of sewage sludge resulted in low organic content, as evident by the high level of ash content compared to aerobic sewage sludge (43% and 27.5%) [99]. In terms of DS, the extraction process was able to lower 3.5% of the organic fraction of the sludge.

Table 8 shows the thermal degradation of DS and RDS. The RDS shows higher thermal stability compared to DS, depicted by higher temperature required to obtain 10, 20, 30, and 40% of mass loss. The thermal stability of the RDS was due to the polymeric 3D floc network [77], which became denser upon dewatering, drying, and further extraction processes. These processes also eliminated water and reduced organic content, leaving a more thermally stable component of the RDS. The RDS showed a higher final residue of 55.51% at 950 °C, suggesting that the sludge particle contains more thermally stable components than DS (48.40%). The higher ash fraction was associated with higher inorganic components in the RDS following extraction [100].

### 3.7. DS and RDS Management and Disposal

Devising the management or disposal of the DS and RDS is highly dependent on its characteristics, in addition to socioeconomic factors [55]. The characteristics of the DS differ from those of other types of sludge, especially the morphological and chemical characteristics, due to the inclusion of polymer flocculant. Raw DS has a high water content along with a low concentration of organic elements, making it impossible to utilize, especially in energy recovery [18]. The extraction process using hexane as a solvent managed to disaggregate surface particles of the DS, as indicated by the formation of smaller particles of debris of around 1.31 ± 0.29 µm. The disaggregation of surface particles is also indicated by the reduction of elements such as P, Na, Mg, and Al, besides O. Despite the reduction, most of the elements still exist in the RDS. The RDS also contained a low organic content of 24.53% fractions. It is evident that there is around 55.51% of a thermally stable fraction of metal oxides and silicate minerals. Aluminosilicate minerals consist of elements such as Si, Al, O, Mg, Na, K, and Fe of various compositions according to the type, including clay and silica [101,102]. Silicate minerals in sand as well as clay are components in the DS and WAS that were trapped during sanitary sewage treatment [99]. The presence of heavy metals in the sludge, such as Fe, originates from the wastewater of small businesses that use metal treatments [99]. The RDS also contained Cl, which resulted from soil mineral dissolution, industrial wastewater discharges, leaching from agricultural soil, and human excreta [99].

A review by Bagheri et al. [55] revealed that there are four dominant sewage sludge management practices based on academic research papers from 1971 to 2019 that include sludge disposal, land application, sludge as a product, and energy recovery. Sludge disposal and land application show a declining trend due to disposal regulations and policies promoting land application as a resource recovery strategy [55]. Resource recovery strategies are divided into material recovery and energy recovery.

There is potential for material recovery from RDS by land application. Land application involves either covering the top layer of soil (mulching) or incorporating the sludge into the soil through ploughing [103]. Applying RDS to the soil by mulching helps reduce evaporation and weed germination, control soil temperature, and minimize nutrient loss [103]. Alternatively, the incorporation of RDS by ploughing can alter the physical properties of the soil, reintroducing the organic matter back to the soil and further increasing the nutrient availability [103]. RDS contains organic fractions of EPSs with phosphorus (P), nitrogen (N), and potassium (K) [104]. P is an essential and irreplaceable component for plants, animals, and humans; thus, recovery and recirculation by land application as fertilizer are necessary for sustainability [105]. Biologically sequestered P remains readily available for biological uptake and may be easily reclaimed upon cellular oxidation [104]. Phosphorous coupled to iron oxides is often not bioavailable to plants and poses challenges for recovery; conversely, potassium bonded to minerals in the soil can be utilized by plants [104]. Noteworthy, the polymer flocculant of PAM also consists of RDS, besides organic matters, and EPSs, which require proper management prior to land application. There are non-specific strategies for treating PAM residue. However, processes such as oxidation, Fenton reactions, and ozone manage to degrade PAM to lower-molecular-weight polymers, releasing EPSs and organic fractions to the soil [77]. Thus, incorporating RDS may alter the characteristics of the soil. Recovery of metal elements such as Fe and Al can also be considered under the material recovery scheme, as around 150,000 tons of Al could be reclaimed in the United States alone [104].

Energy recovery from RDS can be considered through either biological or thermal processes. Thermal processes such as gasification, combustion, incineration, gasification, and pyrolysis, as tabulated in Table 9, have a higher energy potential and are a more suitable option for RDS to generate energy due to the complete elimination of free, interstitial, and vicinal water from the sludge network [2,104]. The possibilities for energy recovery via thermal processes are determined by the objective or product, which in turn influences the process requirements and setup.

However, RDS may contain heavy metals with potential toxicity, which may pose a threat to the environment with land application or land disposal without pretreatment [107]. Therefore, the RDS must be treated prior to land disposal in order to preserve the environment and soil fertility. The pretreatment of RDS can be conducted physiochemically or biologically [108]. Chen et al. [109] treated sewage sludge that contained toxic and harmful substances by solidification treatment with a cementitious binder such as sulfoaluminate cement (SAC). The moisture content of the sewage sludge was reduced by drying and subsequently burned at 440 °C for 6 h to remove organic content [109]. The sewage sludge was added to clay slurry for solidification, and the cementation skeleton formed by the binder hydration products was enhanced due to the exclusion of organic matter [109]. Toxic and harmful matter was stabilized upon completion of treatment, which can be disposed of in a landfill; alternatively, it can be used in the construction industry as subgrade, bricks, or landfill cover [109]. If the RDS from this project is stabilized using this method, the time needed for burning could be reduced due to the lower organic content [109]. Furthermore, drying prior to burning is unnecessary due to the low moisture content. On the other hand, the low moisture content in the RDS makes it suitable for use as raw material in fired clay bricks [89].

### 3.8. Mini Bibliometric Analysis

A mini bibliometric analysis was conducted using the keywords “extraction*” and “sewage sludge” based on the Scopus database. This concise bibliometric study was conducted to give a more thorough insight to the possibilities for research on DS and RDS, especially in the fields of extraction and sustainable sludge management. A total of 2403 publications were compiled between 1973 and 2024, and 2074 articles were evaluated to obtain trends and research hotspots [110,111].

#### 3.8.1. Trend of Publication Related to “Extraction*” and “Sewage Sludge”

**Figure 12:**
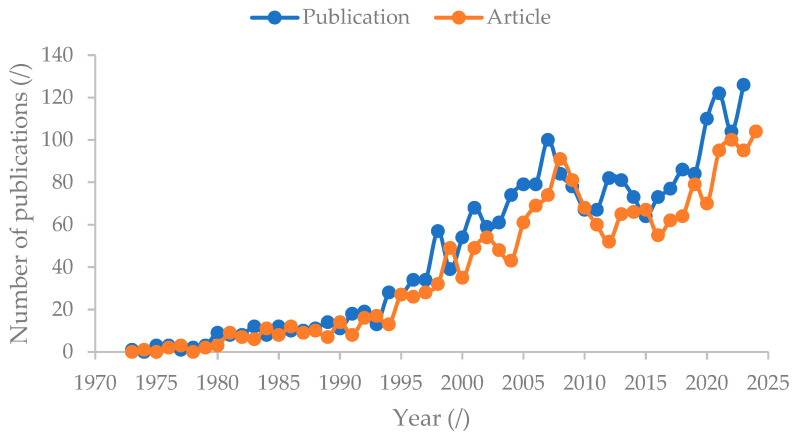
Number of publications on “extraction*” and “sewage sludge”.

Figure 12 shows the increasing trends for publication on “extraction*” and “sewage sludge” from 1973 to 2023. The interest in research related to extraction and sewage sludge publications and articles started in 1973 and gradually increased until 2023. In the current year 2024, a total of 56 publications with 47 articles have been published. The overall increase in publications over the years indicates growing interest in “extraction*” and “sewage sludge”, as well as recognition of their importance. However, fluctuations can be seen from 1998 to 2023, with a major decrease occurring between 2007 and 2011, gradually increasing beyond 2011. The trend was due to government priorities, environmental regulations, and emerging technologies related to sewage sludge management practices [55]. Research related to sewage sludge management decreased in 1990 due to regulations related to sludge disposal and promoting land applications [55]. The usage of sewage sludge as a land application is always associated with contaminants such as heavy metals, organic contaminants, pathogens, and microplastics [55]. The restriction on landfill disposal and land application caused the research of sewage sludge as a product and energy recovery to rise starting from 2009 onwards [55]. Sludge as a product includes sludge-based materials, fertilizer, nitrogen, and phosphorus. Energy recovery is always implemented in combination with the other sludge management practices, focusing on the reduction in organic matter and sludge volume as well as co-treatment [55].

#### 3.8.2. Co-Occurrence Network Analysis

**Figure 13:**
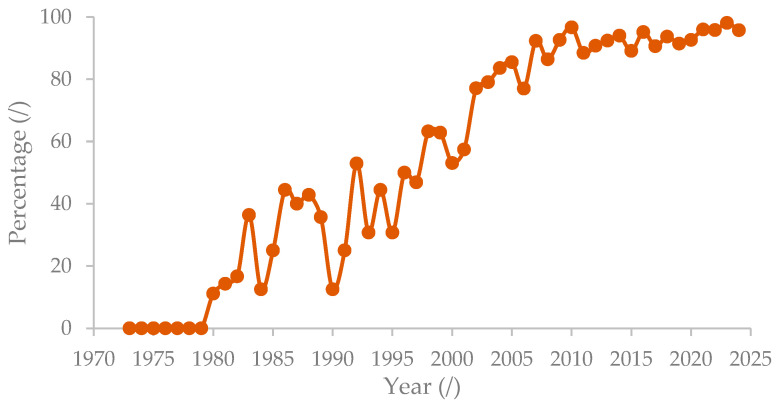
Author keywords usage in articles.

Author keywords were used in the co-occurrence network analysis utilizing VOSviewer. A total of 1672 articles with author keywords were published between 1973 and 2024. The author keywords started to be used in 1980, as presented in Figure 13. There was around 53–97% usage in articles published between 2000 and 2010, and in most of the articles (88–98%) that were published after 2010. There is a total of 4052 keywords based on the published articles, with 252 keywords with a minimum number of five occurrences. The top 30 keywords based on their occurrence are tabulated in Table 10. Sewage sludge, heavy metals, extraction, soil, bioavailability, phosphorus, and compost are among the highest. The use of keywords such as sustainable and circular economy in articles has increased since the United Nations (UN) introduced their Sustainable Development Goals (SDGs) in 2015 [111].

**Figure 14:**
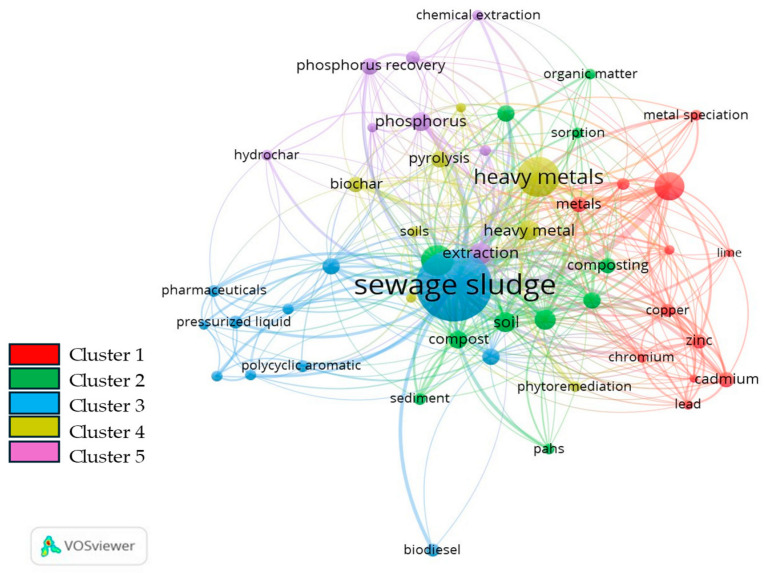
Co-occurrence network of keywords.

Fifty keywords with the highest total link strength (TLS) were selected to provide a broad picture of the research trends from 1980 to 2024 based on clusters [110,111]. Figure 14 shows the co-occurrence network and colored nodes based on clusters. The size of each node indicates the frequency of the keyword’s appearance [112]. The line represents the co-occurrence link between keywords and illustrates the relatedness [113].

The keywords were categorized into five clusters. Sequential extraction, sludge, sewage sludge, heavy metals, and extraction were the most frequently occurring words in each cluster, with 114, 132, 712, 223, and 64 occurrences, respectively. A discussion of keywords based on their various clusters is provided below:

Cluster 1 (Red). Keywords in these clusters are related to the heavy metal speciation and stabilization. Sequential extraction has the largest node with the highest TLS (261) among keywords, followed by zinc (97), cadmium (91), copper (79), metals (75), and leaching (56). Sequential extraction is a leaching technique for analyzing the chemical speciation of heavy metals such as zinc, cadmium, copper, lead, chromium, and nickel from sewage sludge [114]. The extraction and analysis techniques enable an understanding of how these metals are bound within various environmental matrices and further assess their potential mobility and bioavailability [114]. These metals can be released from soils or sediments during leaching processes, leading to environmental contamination and pollution. Additionally, keywords such as stabilization (42) and lime (34) were observed. Lime application is an example of a stabilization technique, where lime is used to immobilize metals by precipitation to prevent their release [115]. This approach is particularly effective in reducing the environmental and health risks associated with the leaching of toxic metals.

Cluster 2 (Green). Keywords in this cluster pertain to sludge and its bioavailability. The keyword with the largest node is sludge with the highest TLS (258), followed by bioavailability (160), soil (157), speciation (118), and compost (116). Bioavailability, in this context, refers to the fraction of contaminants in the sludge or soil, such as polycyclic aromatic hydrocarbons (PAHs), that is accessible to an organism for absorption, the rate of absorption, and its potential toxicity [116]. Composting converts sludge to fertilizer by breaking down the organic content aerobically, which enhances the sorption of PAHs and stabilizes them in the soil [117]. However, high organic content reduced the efficiency of PAH’s removal from sludge by composting [118]. Anaerobic digestion can degrade up to 50% of organic matter; however, no clear disposal method is mentioned regarding the residual sludge [55]. Sludge produced from anaerobic digestion is prone to accumulating a higher content of PAHs compared to aerobic digestion [119].

Cluster 3 (Blue)**.** Keywords in this cluster focus on advanced extraction techniques and resource recovery. Sewage sludge is the keyword with the largest node and highest TLS (363), followed by wastewater (97), biosolid (77), pressurized liquid extraction (57), and polycyclic aromatic hydrocarbon (52). Sewage sludge, as a product of wastewater treatment, was further treated to produce biosolids [120]. Sewage sludge consists of appreciable compounds as well as contaminants [118,121]. Emergence contaminants (EMs) of pharmaceutical products as well as PAHs have the tendency to accumulate in biosolids. These contaminants can be extracted and quantified in order to assess their risk of leaching into the environment. Two keywords related to the advanced technology for solid phase extraction are pressurized liquid extraction (PLE) and microwave-assisted extraction (MAE). Lee et al. [122] optimized MAE for the determination of EMs to replace supercritical fluid extraction (SFE-CO_2_). In this study, Soxhlet extraction was used as the primary reference for the development of MAE techniques [39,122]. They initially compared the performance of MAE with Soxhlet extraction and observed similar results when using the same solvent system [122]. The quantification of contaminants can be analyzed using a chromatography technique coupled with mass spectrometry such as gas chromatography (GC-MS) or liquid chromatography (LC-MS) [122,123]. Additionally, the occurrence of biodiesel (35) among the other keywords indicates an interest in the innovative uses of treated biosolids in biodiesel production, including the extraction of lipids and transesterification [25,42,51], and hydrothermal liquefaction [124]. These technologies offer a sustainable avenue for waste management, specifically in resource recovery for energy production.

Cluster 4 (Yellow). This cluster encompasses keywords associated with contaminants and remediation. Heavy metals and heavy metal displayed the biggest yellow node within this cluster, with TSLs of 498 and 142. The research associated with heavy metals and land application received the highest interest in the 1990s and started decreasing beyond 2000 due to the inclusion of regulations on contaminants and concern for heavy metals [55]. Contaminants such as heavy metals and EMs are associated with biosolids in land application and present significant environmental and health challenges due to their mobility and toxicity in soil [114,123]. Keywords related to remediation such as biochar (88), pyrolysis (86), and phytoremediation (35) were also included in this cluster. Thermal processing of biosolids, including pyrolysis, aims to immobilize or fully destruct the contaminants [120], along with energy recovery and nutrient recycling [55]. Biochar produced from pyrolysis stabilized lead and zinc in soil due to its characteristics that influence metal sorption, thus reducing mobility and bioavailability [125,126]. Incorporation of biochar derived from biosolids enhances the phytoremediation of heavy metals from contaminated soil by white mustard via phytoextraction [126].

Cluster 5 (Purple). This cluster includes the main themes of phosphorus recovery in agricultural application. The largest node belongs to the keyword extraction, followed by phosphorus, phosphorus recovery, and sewage sludge ash, with TSLs of 143, 129, 68, and 50. The interest in research related to these keywords was due to the inclusion of phosphorus in the European Union (EU) crucial raw material listing, price hikes, and the directive to use sewage sludge as fertilizer [55,127]. The extraction of phosphorus in the form of struvite from liquid and solid SS as well as sewage sludge ash represents a crucial advancement in sustainable resource management [128]. Up to 95% phosphorus can be recovered from solid sewage sludge using chemical sequential extraction processes [128]. A single-solvent leaching process using hydrochloric acid (HCl) managed to recover around 60–63% of phosphorus [128]. Keywords such as hydrochar (40) and fertilizer (39) were also included in this cluster. Neha et al. [129] recovered phosphorus from hydrochar produced by hydrothermal liquefaction. Hydrothermal liquefaction is a process of valorizing sewage sludge to biofuel, with hydrochar as a solid residue [129]. The recovered phosphorus as struvite can be processed into high-quality fertilizer, closing the nutrient loop and reducing reliance on conventional phosphorus sources [129]. This integrated approach not only promotes efficient waste utilization but also supports sustainable agriculture by providing a renewable source of a critical nutrient.

## 4. Conclusions

The utilization of sewage sludge for lipid extraction as a raw material for biodiesel offers sustainable avenues for waste management and energy production. Sewage sludge, an end product of wastewater treatment, contains significant organic fractions, including lipids. Dewatered sludge (DS), the most commonly produced type, exhibits unique characteristics, especially in its morphology. This study investigated the influence of particle size on lipid yield, identifying a unimodal distribution of lipid yields for particle sizes <0.5, 0.5–1.0, 1.0–2.0, 2.0–4.0, and >4.0 mm. The highest lipid yield of 1.95 ± 0.56% was achieved from particle sizes 1.0–2.0 mm at 70 °C, 4 h, and a 0.1 g/mL S/L. The structure and morphology of the DS, along with its potential moisture content, influenced the extraction efficiency. The lower extraction yield from DS compared to other types of sludge was due to the lower volatile solid content. Despite extensive research on lipid extraction, the fate of the sludge post-extraction remains unclear. This work characterizes residual dewatered sludge (RDS), revealing reduced water content and organic matter, which minimizes the sludge volume requiring management. Additionally, the high inorganic content improves thermal stability, making the sludge favorable for sustainable reuse. A bibliometric analysis was conducted to provide insights into research trends in “extraction” and “sewage sludge”. The analysis identified five distinct clusters of research: heavy metal speciation and stabilization, sludge and its bioavailability, extraction techniques and resource recovery, contaminant remediation, as well as phosphorus recovery and agricultural applications. Each cluster represents a focused area of investigation within the broader field of sewage sludge utilization and environmental management that can be implemented for DS and RDS. These clusters not only highlight the diverse approaches to studying sewage sludge but also underscore the importance of sustainable practices in waste management and resource recovery.

## Figures and Tables

**Table 1:** Characteristics of several types of sewage sludge.

Types of Sewage Sludge	Ref.	Polymer Flocculant	pH	Moisture Content, MC (%)	Total Solid, TS (wt.%)	Volatile Solid, VS (%)	Total Chemical Oxygen Demand, TCOD (g/kg)
Untreated primary	[20]	No	5.0–8.0	-	2.0–8.0	^a^ 60–80	-
Digested primary	[20]	No	6.5–7.5	-	6.0–12.0	^a^ 30–60	-
Secondary	[20]	No	6.5–8.0	-	0.8–1.2	^a^ 59–88	-
Waste-activated	[21]	No	6.9 ± 0.1	-	^b^ 2.8	^b^ 2.05	^b^ 33.17 ± 0.72
Dewatered	[28]	Yes	-	-	20.2 ± 0.3	14.6 ± 0.1	275.3 ± 7.0
Dewatered	[29]	Yes	-	66.68 ± 1.67	-	35.98 ± 7.95	-
Dewatered	[30]	Yes	7.7 ± 0.1	-	17.1 ± 0.2	^a^ 60.5 ± 0.5	-

^a^ % of TS. ^b^ calculated based on sludge density of 1.07 g/mL [19].

**Table 2:** Content of major volatile solid (biocompounds) in sewage sludge.

Biocompounds	Units	WAS [31]	DS [30]		
			**S-EPS**	**LB-EPS**	**TB-EPS**
Polysaccharides	mg/L	^a^ 506.3–3234	524.30	258.94	1132.06
Proteins	mg/L	2656–13,530	1259.76	1532.24	3708.62
Lipids	mg/L	166–3960	-	-	-
Humic acids	mg/L	196.71–5849	-	-	-

^a^ Carbohydrate. WAS = waste-activated sludge; S = soluble; LB = loosely bound; TB = tightly bound.

**Table 3:** Methods of lipid extraction.

Method	Type of Sludge	Solvent	Lipids Yield (%)	References
Modified Bligh and Dyer	Secondary	Chloroform–methanol	12.6	[46]
Acid hydrolysis	Dewatered	Bromopropane	7.5 ± 0.55	[7]
Water bath shaking	Dewatered	Hexane–ethanol	7.5 ± 0.06	[7]
Boiling extraction (reflux)	Scum Primary Secondary	Methanol–hexane–acetone	33.3 27.0 16.9	[47]
Accelerated solvent extraction system	Secondary	Hexane, methanol	1.94 − 27.43	[48]
Subcritical fluid extraction system	Stabilized (digested)	Liquefied dimethyl ether	2.24	[36]
SFE-CO_2_	Secondary	Carbon dioxide	3.55 − 13.56	[48]
SFE-CO_2_	Sludge cake	Carbon dioxide	0.65	[29]
SFE-CO_2_	Primary	Carbon dioxide H_2_O, C_2_H_5_OH, H_2_O_2_	20.34 − 21.35	[49]
Soxhlet extraction	Dewatered	Hexane–ethanol	10.3 ± 0.20	[7]
Soxhlet extraction	Primary Secondary	Chloroform–methanol	15.6 4.6	[42]
Soxhlet extraction	Primary	Methanol	40.21	[32]
Soxhlet extraction	Sludge cake	Methanol Ethanol	4.05 5.16	[29]
Soxhlet extraction	Primary Secondary Blended Stabilized	Hexane	25.3 9.1 13.9 1	[43,50]
Soxhlet extraction	Dewatered	Methanol	11.05	[51]

**Table 4:** Characteristics of DS.

Parameters	Unit	Value
Moisture content (MC)	%	80.82 ± 0.94
Total solid (TS)	%	19.18 ± 0.94
Volatile solid (VS)	% of TS	46.75 ± 0.74

Values are based on dry weight basis.

**Table 5:** Particle size distribution of DS.

Particle Size (mm)	Percentage (%)
<0.5	13.70 ± 5.87
0.5–1.0	13.58 ± 1.56
1.0–2.0	30.88 ± 4.22
2.0–4.0	25.56 ± 2.70
>4.0	16.29 ± 3.07

**Table 6:** The elemental composition of DS and RDS.

Element	Composition (wt.%)		Difference (wt.%)	
	DS	RDS	Reduce	Increase
O	45.41	36.69	−8.72	
C	23.88	30.79		+6.91
Fe	15.75	18.22		+2.47
P	7.21	4.89	−2.32	
Al	1.90	1.80	−0.10	
Mg	1.35	1.19	−0.16	
S	1.19	1.49		+0.30
Ca	0.98	1.57		+0.59
Si	0.92	2.00		+1.08
Na	0.90	0.62	−0.28	
Cl	0.31	0.41		+0.10
K	0.21	0.32		+0.11

−: reduce; +: increase.

**Table 7:** Mass loss of DS, RDS, WAS, and polymer flocculant.

Sample	Mass Loss at Temperature (%)					Residue at 950 °C (%)
	150 °C	202 °C	210 °C	450 °C	491 °C	
WAS	5.27	6.09	6.24	29.92	34.06	35.34
Polymer flocculant	12.18	15.72	16.18	71.21	72.53	1.66
DS	3.52	6.09	6.44	31.63	34.06	48.40
RDS	0.23	0.42	0.48	22.41	24.95	55.51

**Table 8:** Thermal degradation of DS and RDS.

Sludge	Temperature of Mass Loss (°C)			
	10%	20%	30%	40%
DS	252.83	330.50	428.17	683.50
RDS	323.00	418.17	638.00	840.17

**Table 9:** Thermochemical process of sewage sludge [2,104,106].

Technology	Objectives	Process	Product/Emission
Gasification	Conversion of organic materials and inert material Energy recovery (heat)	800–900 °C with limited oxygen Gasifying media	Syngas (H_2_, CO, CO_2_ and hydrocarbons) Gasification ash
Combustion	Burning organic materials Nutrient recovery Energy recovery (heat)	Burning organic materials in the presence of excess air	Gases (CO_2_, CO, H_2_O, NO_x_, SO_x_, VOCs) Fly ash Particulate matter
Incineration	Volume reduction Harmful substances destruction Energy recovery (heat)	>760 °C in the presence of excess air	Gases (CO_2_, CO, H_2_O, NO_x_, SO_x_, VOCs) Incinerator ash Particulate matter
Pyrolysis	Thermal decomposition of organic material Energy recovery (heat and materials)	300–700 °C in absence of oxygen	Liquid pyrolytic oil (bio-oil) Solid biochar Non-condensable gas

**Table 10:** Top 30 keywords on “extraction*” and “sewage sludge”.

No	Keyword	Cluster	Occurrences	TLS
1	Sewage sludge	3	712	1363
2	Heavy metals	4	223	498
3	Sludge	2	132	258
4	Sequential extraction	1	114	261
5	Extraction	5	64	143
6	Soil	2	61	157
7	Heavy metal	4	61	142
8	Bioavailability	2	56	160
9	Phosphorus	5	50	129
10	Compost	2	46	116
11	Anaerobic digestion	2	42	66
12	Wastewater	3	42	97
13	Phosphorus recovery	5	42	68
14	Speciation	2	40	118
15	Pyrolysis	4	40	86
16	Biosolids	3	38	77
17	Metals	1	36	75
18	Cadmium	1	35	91
19	Biochar	4	35	88
20	Composting	2	33	83
21	Zinc	1	29	97
22	Sewage sludge ash	5	26	50
23	Copper	1	25	79
24	Pressurized liquid extraction	3	24	57
25	Biodiesel	3	24	35
26	Leaching	1	23	56
27	Pharmaceuticals	3	23	48
28	Polycyclic aromatic hydrocarbon	3	22	52
29	Sediment	2	20	44
30	Microwave-assisted extraction	3	19	49

TLS: total link strength.

## Data Availability

The original contributions presented in the study are included in the article, further inquiries can be directed to the corresponding author.
